# Cancer treatments-related factors for subsequent soft-tissue sarcoma in childhood cancer survivors

**DOI:** 10.1016/j.esmoop.2026.108313

**Published:** 2026-07-24

**Authors:** R.S. Allodji, R.C. Reulen, I. Diallo, D.L. Winter, G. Vu-Bezin, S. Bolle, M. Locquet, F. Bagnasco, E. Bárdi, E.A.M. Feijen, D. Alessi, M.M. Fidler-Benaoudia, S. Høgsholt, C.J. Bright, H. Linge, B. Fresneau, N. Haddy, C. Veres, D. Llanas, N. Journy, C. Demoor-Goldschmidt, J. Byrne, D. Bejarano-Quisoboni, D. Grabow, W. Zrafi, T. Gudmundsdottir, G. Michel, W. Gunnes, P. Kaatsch, C. Rubino, H. Jenkinson, M. Kaiser, R. Skinner, R. Aho Glele, C. Ducos, N. Aba, T. Cole, N. Waespe, S. Nordenfelt, T. Charrier, M. Zidane, M. Jankovic, T. Lähteenmäki, M.M. Maule, H.J.H. van der Pal, C.M. Ronckers, F.E. van Leeuwen, J. Teepen, M. Terenziani, T. Wiebe, C. Sacerdote, Z. Jakab, R. Haupt, P.M. Lähteenmäki, L. Zadravec Zaletel, C.E. Kuehni, J.F. Winther, L.C. Kremer, L. Hjorth, M.M. Hawkins, F. de Vathaire

**Affiliations:** 1 Université Paris-Saclay, Gustave Roussy, Inserm, UVSQ, Center for Research in Epidemiology and Population Health – Unit 1018, EpiRad (Radiation Epidemiology team), Villejuif, France; 2 Centre for Childhood Cancer Survivor Studies, School of Health Sciences, University of Birmingham, Birmingham, UK; 3 Université Paris-Saclay, Gustave Roussy, INSERM U1030, Molecular Radiation Therapy and Therapeutic Innovation, Villejuif, France; 4 Centro de Protonterapia Quironsalud, Pozuelo de Alarcon, Madrid, Spain; 5 Scientific Directorate, IRCCS Istituto Giannina Gaslini, Genova, Italy; 6 St Anna Children’s Hospital, Vienna, Austria; 7 Department of Paediatrics and Adolescent Medicine, Johannes Kepler University Linz, Kepler University Hospital, Linz, Austria; 8 Princess Máxima Center for Pediatric Oncology, Utrecht, The Netherlands; 9 Childhood Cancer Registry of Piedmont, Cancer Epidemiology Unit, Department of Medical Sciences, University of Turin and CPO-Piemonte, AOU Città della Salute e della Scienza di Torino, Torino, Italy; 10 Cancer Epidemiology and Prevention Research, Alberta Health Services, Calgary, Alberta; 11 Departments of Oncology and Community Health Sciences, University of Calgary, Calgary, Alberta, Canada; 12 Danish Cancer Society Research Center, Childhood Cancer Research Group, Copenhagen; 13 Department of Pediatrics, Aarhus University Hospital, Aarhus, Denmark; 14 NHS Digital, Leeds, UK; 15 Department of Clinical Sciences Lund, Division of Pediatrics, Lund University, Skane University Hospital, Lund, Sweden; 16 Boyne Research Institute, Bettystown, Ireland; 17 German Childhood Cancer Registry, Division of Childhood Cancer Epidemiology, Institute of Medical Biostatistics, Epidemiology and Informatics (IMBEI), Johannes-Gutenberg University Mainz, Germany; 18 Childreńs Hospital, Landspitali University Hospital, Reykjavik, Iceland; 19 Faculty of Health Sciences and Medicine, University of Lucerne, Lucerne, Switzerland; 20 Norwegian National Advisory Unit on Solid Tumors in Children, Oslo, Norway; 21 Norwegian Cancer Registry and Department of Pediatric Medicine, Oslo University Hospital and Institute of Clinical Medicine, Faculty of Medicine, University of Oslo, Oslo, Norway; 22 Birmingham Children’s Hospital, Birmingham, UK; 23 Great North Children’s Hospital, Newcastle upon Tyne Hospitals NHS Foundation Trust, and Newcastle University Centre for Cancer, Newcastle University, Newcastle upon Tyne, UK; 24 Autorité de Sûreté Nucléaire et de Radioprotection (ASNR), PSE-SANTE/SERAMED/LRMED, Fontenay-aux-Roses, France; 25 West Midlands Regional Genetics Laboratory, Birmingham Women’s Hospital, Birmingham, UK; 26 CANSEARCH Research Platform for Paediatric Oncology and Haematology, Department of Paediatrics, Gynaecology and Obstetrics, Faculty of Medicine, University of Geneva, Geneva, Switzerland; 27 Childhood Cancer Research Group, Institute of Social and Preventive Medicine, University of Bern, Bern, Switzerland; 28 Division of Pediatric Hematology/Oncology, Department of Pediatrics, University Children’s Hospital of Bern, University of Bern, Bern, Switzerland; 29 Lund University Cancer Centre, Lund, Sweden; 30 Pediatric Clinic, University of Milano-Bicocca, Hospital San Gerardo, Monza, Italy; 31 Department of Pediatrics and Adolescent Medicine, Turku University and Turku University Hospital, Turku, Finland; 32 Division of CAYA Cancer Survivorship Research, German Cancer Research Center, Heidelberg, Germany; 33 Department of Epidemiology, Division of Psychosocial Research & Epidemiology, Netherlands Cancer Institute, Amsterdam, The Netherlands; 34 Pediatric Oncology Unit, Fondazione IRCCS Istituto Nazionale dei Tumori, Milano, Italy; 35 Hungarian Childhood Cancer Registry, 2^nd^ Department of Pediatrics, Semmelweis University, Budapest, Hungary; 36 DOPO Clinic, Division of Hematology/Oncology, IRCCS Istituto Giannina Gaslini, Genova, Italy; 37 Division of Radiotherapy, Institute of Oncology, Ljubljana, Slovenia; 38 Medical Faculty, University of Ljubljana, Ljubljana, Slovenia; 39 Department of Clinical Medicine, Faculty of Health, Aarhus University and University Hospital, Aarhus, Denmark; 40 Department of Pediatric Oncology, Emma Children’s Hospital & Academic Medical Center, Amsterdam, The Netherlands

**Keywords:** chemotherapy, childhood cancer survivors, procarbazine, radiation therapy, subsequent soft-tissue sarcoma

## Abstract

**Background:**

Previous studies of risk factors for subsequent soft-tissue sarcoma (STS) among childhood cancer survivors had small numbers and were unable to comprehensively investigate the dose–response relationships with radiation from radiotherapy and with cumulative exposure to specific cytotoxics.

**Patients and methods:**

We conducted a nested case-control study, encompassing 275 subsequent STS cases and 275 matched controls, within the Pan-European cohort of 69 460 5-year survivors from 12 countries. Odds ratios (ORs) and 95% confidence intervals (CIs) for subsequent STS were calculated for different levels of radiation dose to the STS location (in Gy) and for cumulative doses of specific chemotherapeutic agents (in g/m^2^). Additionally, excess ORs per Gy (EOR/Gy) or per g/m^2^ (EOR/g/m^2^) were calculated to assess dose–response relationships.

**Results:**

The OR for subsequent STS was 22-fold [95% CI 6.9-95.4] higher in soft tissue exposed to ≥30 Gy and remained in excess with exposure to 5-9 Gy [OR = 3.8, 95% CI 1.3-12.0] compared with no radiation. The EOR/Gy was 0.86 [95% CI 0.35-2.16], with a particularly high risk observed in survivors of neuroblastoma [EOR/Gy = 5.52, 95% CI 0.71-52.31] or bone sarcoma [EOR/Gy = 4.16, 95% CI 0.40-24.72], and in females [EOR/Gy = 2.35; 95% CI 0.65-8.14]. For patients who had received a cumulative procarbazine dose of ≥6.0 g/m^2^, the OR was 4.7 [95% CI 1.3-25.1] compared with non-exposure after controlling for radiation. No association was found for other alkylating agents or other specific cytotoxic drugs.

**Conclusion:**

Although high radiation doses remain the primary risk factor for secondary STS, our findings suggest a possible increase in risk at lower doses (5-9 Gy) and following procarbazine treatment among childhood cancer survivors. These observations warrant further investigation and may merit consideration in treatment planning and long-term follow-up guidelines for cancer survivors.

## Highlights

• The largest nested case-control study of 275 subsequent STS in childhood cancer survivors.
• Subsequent STS risk increased with radiation dose, with elevated risk observed even at low-to-moderate doses of 5-9 Gy.
• Subsequent STS radiation-related risk was highest in neuroblastoma, bone sarcoma survivors, and females.
• Procarbazine exposure was independently associated with increased subsequent STS risk.
• Findings warrant further study and may inform treatment planning and long-term follow-up of childhood cancer survivors.

## Introduction

The number of childhood cancer survivors is anticipated to grow due to further improvements in childhood cancer treatments and care,^1^ and a slow increase in childhood cancer incidence.^2^ Accordingly, awareness of and insight into side-effects and long-term consequences of childhood cancer treatments have become increasingly important.^3^ Indeed, significant long-term morbidities continue to affect the majority of childhood cancer survivors.^1^^,^^3^ One of the most life-threatening sequelae among childhood cancer survivors is the occurrence of subsequent primary neoplasms (SPN), which are defined as histologically distinct tumours developing after primary cancer therapy.^4^, ^5^, ^6^, ^7^ The occurrence of an SPN may be influenced by prior anticancer treatments, individual genetic susceptibility, as well as host and lifestyle factors. Radiotherapy and chemotherapy, particularly alkylating agents, platinum-based drugs, or topoisomerase II inhibitors, may have carcinogenic effects.^6^^,^^7^ Preexisting genetic conditions, such as hereditary retinoblastoma (e.g. due to *RB1* germline pathogenic variants), also predispose patients to multiple primary tumours.^8^

An increased risk of subsequent soft-tissue sarcoma (STS) has been found in survivors of various types of childhood cancer, including retinoblastoma, central nervous system tumours, Hodgkin lymphoma, bone sarcoma, Wilms’ tumour, and leukaemia.^9^, ^10^, ^11^ Previous reports have noted 2- to 16-fold increased risks of subsequent STS following 5-year survival of childhood cancer,^9^^,^^11^ relative to the general population. However, because development of a subsequent STS is rare, the few previous reports on subsequent STS risk factors were based on small observed numbers of 32, 23, 53, and <70 subsequent STS cases.^12^, ^13^, ^14^, ^15^ These previous studies indicated that radiotherapy and chemotherapy treatments increase the risk of subsequent STS among childhood cancer survivors. Nevertheless, many questions remain about these relationships, particularly regarding the nature of the dose–response relationships at low-to-moderate (<10 Gray, Gy) and very high (≥30 Gy) absorbed doses of therapeutic radiation to muscle or soft tissue. Furthermore, the potential impact of the reduction of therapeutic radiation exposures in recent treatment protocols for childhood cancers on the subsequent STS risk has not been investigated. Moreover, the potential role of chemotherapy in subsequent STS risk, either with or without radiotherapy, has not been adequately explored. Although previous studies have suggested an increased subsequent STS risk associated with anthracyclines and alkylating agents,^12^, ^13^, ^14^, ^15^ these studies were unable to disentangle risks associated with specific agents because of the small numbers of subsequent STS cases observed.

In this study, we investigate the clinical and therapeutic factors associated with the long-term risk of subsequent STS, including anticancer treatment modalities and the corresponding cumulative exposure doses, in a large case-control study of 275 cases of subsequent STS ascertained and 275 matched controls, nested within the collaborative Pan-European PanCareSurFup cohort study from 12 European countries.

## Patients and methods

### The nested case-control study within the PanCareSurFup cohort

Patients were selected from the PanCareSurFup cohort, which comprises 69 460 5-year childhood cancer survivors who were diagnosed before age of 20 between 1940 and 2008. This cohort comprises data from both population-based cancer registries and major treatment centres across 13 European cohorts within 12 countries.^16^ It has been previously described and includes data from population-based cancer registries and major treatment centres. Ethical approval was obtained for all contributing subcohorts.^9^^,^^17^, ^18^, ^19^

### Ascertainment of subsequent STS cases

SPNs were ascertained through several different methods, including population-based cancer registries (over 67% of cases),^9^ medical and hospital data, questionnaires for survivors, long-term follow-up clinics, health insurance databases, and national mortality records. Validation relied primarily on pathology reports, or when unavailable, documented clinical diagnoses as previously described.^9^^,^^17^, ^18^, ^19^ A total of 275 childhood cancer survivors were diagnosed with a subsequent STS at least 5 years after their first primary neoplasm (FPN).

### Matching process

Each subsequent STS case was matched to one control on subcohort, sex, age at FPN (±1 year), calendar year of FPN (±3 years), and retinoblastoma status (heritable/non-heritable) using density sampling. To avoid overmatching on treatment, FPN type itself other than heritable retinoblastoma was not used as a matching factor. Retinoblastoma was considered heritable when bilateral disease, family history, or both were present.^20^^,^^21^

Controls were required to have a follow-up duration longer than the time to subsequent STS in the matched case.

### Data collection

For each individual, detailed information was collected on clinical characteristics, radiotherapy plans, and cumulative doses of anticancer treatments received during the interval between the diagnosis of the FPN and (i) subsequent STS for the case and (ii) the corresponding interval in the matched control.

### Radiation dosimetry

Radiotherapy simulation files and treatment charts were obtained for all irradiated participants. Radiation doses to the anatomic site where each case developed STS and the corresponding site in the matched control were reconstructed with voxel-based anthropomorphic phantoms individualized for sex, age, height, weight, and treatment position. Because whole-body computed tomography based planning was not available historically, treatments were reconstructed using the ISOgray system. STS locations were determined from pathology, surgical, and diagnostic reports. For each case, contours of the STS volume were created in the phantom; identical anatomic contours were defined for matched controls. We used the mean dose to these contours as the primary dosimetric measure. Dosimetry reconstruction methods have been reported previously.^22^

### Chemotherapy exposure

Medical records were reviewed to document all cytotoxic agents administered, including drug names, dates, and total doses per square meter (mg/m^2^). Cumulative doses (mg/m^2^) for each drug were calculated across all cycles. Chemotherapy drugs were categorised into classes: alkylating agents, anthracyclines, epipodophyllotoxins, platinum compounds, vinca alkaloids, and antimetabolites (Supplementary Table S1, available at https://doi.org/10.1016/j.esmoop.2026.108313). Alkylating agent dose was expressed as cyclophosphamide equivalent dose.^23^ Associations were examined for cumulative dose categories for each drug class and, when ≥30 participants were exposed, for individual agents.

### Statistical analysis

Conditional logistic regression estimated odds ratios (ORs) and 95% confidence intervals (CIs) for subsequent STS. Radiation exposure at the STS site was categorised as follows: 0, <5, 5-9, 10-19, 20-29, and ≥30 Gy. For each category, we calculated attributable risk (AR) among those exposed as AR = 100 × (OR − 1)/OR, representing the proportion of cases among exposed individuals that is statistically attributable to the exposure. The population attributable risk (PAR) was calculated as PAR = AR × *Pe*, where *Pe* denotes the proportion of cases in the population who are exposed; PAR thus represents the proportion of cases in the overall population that is statistically attributable to the exposure.^24^ The 95% CI for AR and PAR were estimated using the Delta method. These estimates are based on several assumptions, including the existence of a causal link between exposure and effect, the absence of residual confounding factors, and the correct specification of the dose–response model. Given the observational nature of the present study, these assumptions cannot be fully verified, and this should be borne in mind when interpreting the results.^25^ Ordinal categories were modelled continuously to test for trend. Additionally, to assess the dose–response relationship, we compared linear (EOR(d)=1+βd), linear-quadratic ($EOR\left(d\right)=1+\beta d+{\gamma d}^{2}$), and linear-quadratic-cubic models ($EOR\left(d\right)=1+\beta d+\gamma d^{2}+\emptyset d^{3}$), where d is the radiation exposure at the STS site and β*, Ɣ and ɸ* represents the excess odd ratio (EOR) per Gy, Gy2 and per Gy3, respectively. Departure from linearity was evaluated using likelihood ratio tests. In the absence of statistically significant improvement in model fit, the linear model was retained for its parsimony and interpretability. Effect modification of the EOR/Gy was evaluated by sex, FPN type, age at FPN diagnosis, decade of FPN diagnosis, attained age, time since FPN, STS histological type, and exposure to chemotherapy (alkylating agents, procarbazine, and anthracyclines). Associations between cumulative chemotherapy dose (by class and by specific drugs) and STS were examined using similar conditional logistic regression methods.^18^^,^^19^ Sensitivity analyses excluded retinoblastoma survivors (leaving 219 case-control pairs) and then restricted to retinoblastoma survivors only (leaving 56 pairs). All analyses were carried out using SAS 9.3^26^ and Epicure software,^27^ with a significant threshold of *P* < 0.05.

## Results

### Cases and controls characteristics

Of 275 patients who had subsequent STS, 155 were males, retinoblastoma was a prior diagnosis in 56 (20.4%; Table 1), while only 7% had STS as their FPN. Among cases, the median age at FPN diagnosis was 5 years (range 0.1-19.7 years); diagnoses occurred between 1944 and 2001. The median attained age at subsequent STS diagnosis was 28.6 years (range 6.0-62.1), which occurred a median of 20.4 years (range 5.2-61.6) after their FPN. Among cases, the shortest median interval to subsequent STS was for survivors of Hodgkin lymphoma (13 years), contrasting with the longest interval of 36 years among retinoblastoma survivors (Supplementary Table S2, available at https://doi.org/10.1016/j.esmoop.2026.108313). Among cases, 47 (17.2%) patients had heritable retinoblastoma, 8 (2.8%) had non-heritable retinoblastoma, and 1 (0.4%) had retinoblastoma with unknown hereditary status; similar proportions were observed in the control group (Table 1). The most commonly observed subsequent STS subtypes were myomatous neoplasms (*n* = 86, 31.3%), fibromatous neoplasms (*n* = 42, 15.3%), nerve sheath tumours (*n* = 41, 14.9%), and STS not otherwise specified (*n* = 53, 19.3%; Table 1). Approximately 25% of subsequent STS were located in the breast, uterus, and pelvic areas.

**Table 1:** Demographics and clinical characteristics of cases and matched controls included in the nested case-control study of soft tissue sarcoma risk among childhood cancer survivors

Characteristics	Patients with a subsequent STS^b^ (*n* = 275)	Controls (*n* = 275)
Sex^a^		
Female	120 (43.6)	120 (43.6)
Male	155 (56.4)	155 (56.4)
FPN diagnosis		
Leukaemia	25 (9.1)	31 (11.3)
Hodgkin lymphoma	31 (11.3)	24 (8.7)
Non-Hodgkin lymphoma	7 (2.5)	15 (5.5)
Central nervous system	39 (14.2)	44 (16)
Neuroblastoma	16 (5.8)	8 (2.9)
Retinoblastoma	56 (20.4)	56 (20.4)
Wilms tumour	31 (11.3)	25 (9.1)
Bone sarcoma	21 (7.6)	11 (4)
STS	20 (7.3)	29 (10.5)
Other and not classifiable	29 (10.5)	32 (11.6)
Age at diagnosis of FPN (years)^a^		
*Median (min-max)*	*5 (0.1-19.7)*	*5 (0.1-19.7)*
<1	47 (17.1)	39 (14.2)
1-3	76 (27.6)	82 (29.8)
4-7	44 (16)	46 (16.7)
8-11	35 (12.7)	34 (12.4)
12-20	73 (26.5)	74 (26.9)
Decade of diagnosis of FPN^a^		
*Median (min-max)*	*1976 (1944-2001)*	*1975 (1945-2001)*
<1970	100 (36.4)	100 (36.4)
1970-1979	72 (26.2)	71 (25.8)
1980-1989	73 (26.5)	76 (27.6)
≥1990	30 (10.9)	28 (10.2)
Retinoblastoma status^a^		
Heritable	47 (17.2)	47 (17.2)
Nonheritable	8 (2.8)	8 (2.8)
Retinoblastoma, but status unknown	1 (0.4)	1 (0.4)
Other type of childhood cancer	219 (79.6)	219 (79.6)
Radiotherapy (RT)		
No	46 (16.7)	91 (33.1)
Yes	222 (80.7)	177 (64.4)
Missing	7 (2.5)	7 (2.5)
Chemotherapy (CT)		
No	104 (37.8)	134 (48.7)
Yes	166 (60.4)	134 (48.7)
Missing	5 (1.8)	7 (2.5)
Combination of treatment of FPN		
Neither RT nor CT	20 (7.3)	57 (20.7)
RT only	82 (29.8)	77 (28)
CT only	26 (9.5)	34 (12.4)
Both RT and CT	140 (50.9)	100 (36.4)
Missing	7 (2.5)	7 (2.5)
Vital status at end of follow-up		
Alive	102 (37.1)	246 (89.5)
Dead	173 (62.9)	29 (10.5)
Subsequent STS^b^ by histological type		
Fibromatous neoplasms	42 (15.3)	
Lipomatous neoplasms	22 (8)	
Myomatous neoplasms: *[Leiomyosarcoma (n = 72)]*	86 (31.3)	
Nerve sheath tumours	41 (14.9)	
Soft-tissue tumours and sarcomas, NOS	53 (19.3)	
Others STS^b^	31 (11.3)	
Subsequent STS^b^ location		
Breast, uterine and pelvic area	68 (24.7)	
Other parts of the body	207 (75.3)	
Attained age at subsequent STS^b^ (years)		
*Median (min-max)*	*28.6 (6-62.1)*	
5-14	31 (11.3)	
15-24	75 (27.3)	
25-34	88 (32)	
35-44	50 (18.2)	
≥45	31 (11.3)	
Years from FPN diagnosis (years)^c^		
*Median (min-max)*	*20.4 (5.2-61.6)*	
5-9	45 (16.4)	
10-19	91 (33.1)	
20-29	67 (24.4)	
30-39	41 (14.9)	
≥40	31 (11.3)	

a Matching criteria.

b STS, soft-tissue sarcoma; min, minimum; max, maximum; FPN, first primary neoplasm.

c Years from FPN diagnosis, years between FPN diagnosis and STS diagnosis; others STS, [vascular and perivascular sarcomas (*n* = 10), synovial-like neoplasms (*n* = 8), Ewing sarcoma (*n* = 4), malignant mesenchymoma (*n* = 3), primitive and peripheral neuroectodermal tumours (*n* = 3), neurilemmoma malignant (*n* = 2), and histiocytic sarcoma (*n* = 1)].

### Radiation dose–response relationships for subsequent STS

**Figure 1:**
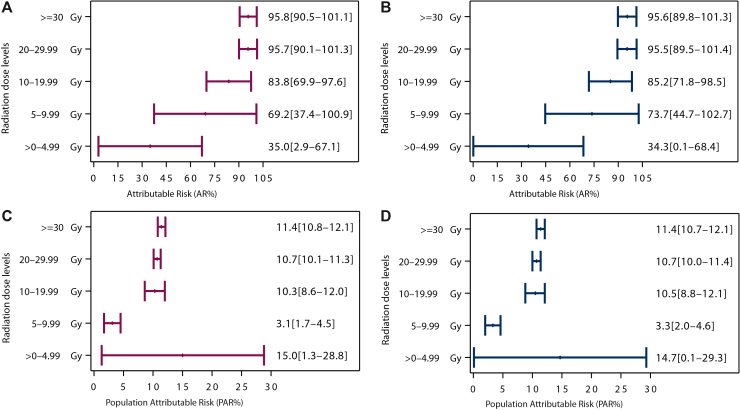
AR% and PAR% of subsequent soft-tissue sarcoma attributable to exposure to dose of radiation (in Gy) received to site of soft-tissue sarcoma, with (purple) or without (blue) adjustment for procarbazine exposure. AR%, attributable risk; PAR%, population attributable risk.

The majority of cases (80.7%) and controls (64.4%) had received radiotherapy (Table 1). Cases had average cumulative radiation doses at the STS site twice that of controls (13.2 Gy versus 4.8 Gy). The odds of developing subsequent STS increased with increasing cumulative local radiation dose levels: 5-9, 10-19, 20-29, and 30+ Gy were associated with ORs of 3.8 [95% CI 1.3-12.0], 6.7 [95% CI: 2.9-17.5], 22.1 [95% CI 6.7-94.7], and 22.6 [95% CI 6.9-95.4], respectively, after adjustment for cumulative exposure to procarbazine (Table 2). The corresponding ARs were 73.7%, 85.2%, 95.5%, 94%, and 95.6%, respectively, while the PARs were 14.7%, 3.3%, 10.5%, 10.7%, and 11.4%, respectively (Figure 1). For example, ∼11% of STS cases were statistically attributable to exposure to radiation doses ≥30 Gy at the population level (PAR), whereas among exposed individuals, up to 96% of STS cases were statistically attributable to this exposure (AR), under the assumptions of the model. Similar trends were observed when excluding retinoblastoma survivors, although CIs widened (Table 2). When fitting continuous cumulative radiation dose as a linear term, the EOR per Gy was 0.86 [95% CI 0.35-2.16] (Supplementary Table S3 and Figure S1, available at https://doi.org/10.1016/j.esmoop.2026.108313). Nonlinear models did not improve fit (*P* = 0.80; Supplementary Table S3, available at https://doi.org/10.1016/j.esmoop.2026.108313). While the EORs per Gy were generally consistent across subgroups (Supplementary Table S4, available at https://doi.org/10.1016/j.esmoop.2026.108313), significant heterogeneity was observed for sex [*P*_interaction_ = 0.02, with a higher EOR per Gy of 2.35 (95% CI 0.65-8.14) for females versus EOR per Gy of 0.33 (95% CI 0.12-0.93) for males], FPN diagnosis [*P*_interaction_ = 0.02, with higher EORs per Gy of 5.52 (95% CI 0.71-52.31) and 4.16 (95% CI 0.40-24.72) in neuroblastoma and bone sarcoma survivors, respectively], and attained age [*P*_interaction_ = 0.01, with higher EOR per Gy of 0.97 (95% CI 0.15-5.36) in survivors aged ≥35 years and 0.81 (95% CI 0.15-5.36) in those aged <20 years].

**Table 2:** Risk of subsequent soft-tissue sarcoma in relation to cumulative dose of radiation received to site of soft-tissue sarcoma adjusted for procarbazine

	Dose category of radiation (Gy)	Mean dose (Gy) in cases/controls	Mean years to STS	Cases/controls	Cumulative radiation dose	Cumulative radiation dose + adjusted for procarbazine
					OR^b^ [95% CI]	OR^b^ [95% CI]
All survivors (*n* = 550)	0		19.2	46/91	1.0 (Reference)	1.0 (Reference)
	>0-4.99	0.9/0.5	26.9	115/136	1.5 [0.9-2.5]	1.5 [0.9-2.6]
	5-9.99	7.6/7.7	25.0	12/12	3.2 [1.2-9.4]	3.8 [1.3-12.0]
	10-19.99	14.4/14.6	17.8	33/16	6.2 [2.8-15.2]	6.7 [2.9-17.5]
	20-29.99	24.4/26.3	19.5	30/7	23.1 [7.2-96.8]	22.1 [6.7-94.7]
	≥30	42.3/38.9	18.3	32/6	24.0 [7.7-98.3]	22.6 [6.9-95.4]
	Missing		26.6	7/7		
	*P-value for trend*				*<0.0001*	*<0.0001*
All survivors excluding retinoblastoma (*n* = 438)	0		17.2	40/79	1.0 (Reference)	1.0 (Reference)
	>0-4.99	1.2/0.5	21.7	75/102	1.4 [0.8-2.4]	1.3 [0.7-2.4]
	5-9.99	8.1/7.6	19.6	9/9	4.1 [1.2-16.1]	5.6 [1.5-26.8]
	10-19.99	14.5/14.3	17.1	31/14	6.1 [2.6-16.4]	6.9 [2.7-20.1]
	20-29.99	24.4/27.1	19.6	28/6	35.8 [8.9-228.6]	34.9 [8.3-230.9]
	≥30	42.6/38	18.6	30/5	37.9 [9.7-230.8]	37.1 [9.1-233.7]
	Missing		24.1	6/4		
	*P-value for trend*				*<0.0001*	*<0.0001*
Retinoblastoma survivors (*n* = 112)^a^	0		37.7	6/12	1.0 (Reference)	
	>0-4.99	0.4/0.4	36.4	40/34	2.4 [0.8-8.8]	
	5-9.99	6/8.2	32.7	3/3	1.8 [0.3-11.8]	
	≥10	25.5/24.3	17.3	6/4	3.9 [0.6-29.2]	
	Missing		39.6	1/3		
	*P-value for trend*				*0.2*	

95% CI, 95% confidence interval; OR, odds ratio.

a None retinoblastoma survivors received procarbazine; therefore, only results from the unadjusted model are presented.

b Conditional logistic regression matched on sex, age at childhood cancer (±1 year), calendar year of childhood cancer diagnosis (±3 years), and retinoblastoma status (heritable/non-heritable).

### Cytotoxic chemotherapy dose–response relationships for subsequent STS

**Figure 2:**
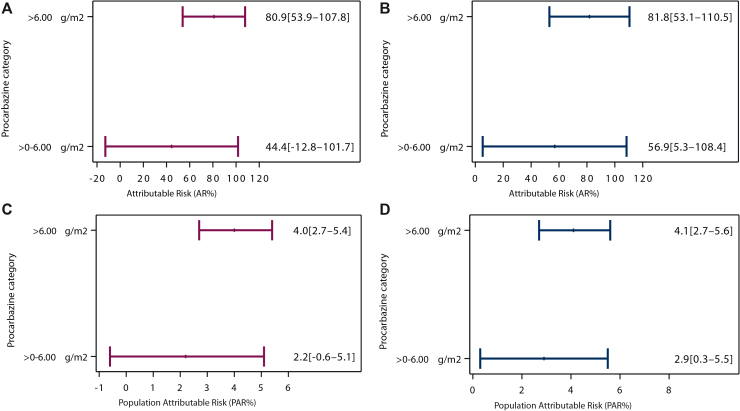
AR% and PAR% of subsequent soft-tissue sarcoma attributable to exposure to procarbazine dose (in g/m^2^), with (purple) or without (blue) adjustment for dose of radiation. AR%, attributable risk; PAR%, population attributable risk.

Table 3 shows our assessment of the potential explanatory role of cumulative exposure to each class of cytotoxic agents on the risk of subsequent STS. The only class with evidence of impact was the alkylating agents (*P* = 0.02). Table 3 considers the potential explanatory power of cumulative exposure to each specific cytotoxic agent with at least 30 cases or controls exposed—procarbazine was the only specific cytotoxic agent with evidence of impact on the risk of subsequent STS (*P* < 0.001). Notably, cumulative exposure to all alkylating agents except procarbazine was not predictive of subsequent STS risk (*P* = 0.10, Table 3). After controlling for cumulative radiation dose, elevated risk of subsequent STS was observed among patients who received a cumulative procarbazine dose of at least 6.0 g/m^2^ with an OR = 4.7 [95% CI 1.3-25.1] compared with those who did not receive procarbazine (Table 3). This increased risk remained also unchanged after excluding retinoblastoma survivors [OR = 4.8 (95% CI 1.3-26.2); Supplementary Table S5, available at https://doi.org/10.1016/j.esmoop.2026.108313]. Most patients treated for Hodgkin lymphoma had been exposed to procarbazine at cumulative doses ≥6.0 g/m^2^ (Supplementary Table S6, available at https://doi.org/10.1016/j.esmoop.2026.108313). Approximately 4% of STS cases in the overall population were statistically attributable to procarbazine exposure at cumulative doses ≥6.0 g/m^2^, while 81.8% of cases among exposed patients were statistically attributable to this exposure under the model assumptions (Figure 2). There was no evidence of a departure from linearity between continuous cumulative procarbazine dose and subsequent STS risk [EOR/g/m^2^ = 0.46 (95% CI 0.01-2.66); Supplementary Table S7, available at https://doi.org/10.1016/j.esmoop.2026.108313]. Due to model convergence issues, higher-order effects (quadratic and cubic) could not be assessed. Supplementary Table S8, available at https://doi.org/10.1016/j.esmoop.2026.108313 presents potential procarbazine effect modifiers. While no significant heterogeneity was observed across most subgroups, the dose–response for procarbazine varied depending on the radiation dose. A significant procarbazine dose–response relationship was evident among survivors exposed to <20 Gy of radiation [EOR/g/m^2^ = 0.39 (95% CI 0.03-2.11)].

**Table 3:** Risk of subsequent soft-tissue sarcoma in relation to cumulative exposure to specific types of chemotherapy adjusted for dose of radiation received to site of soft-tissue sarcoma

Chemotherapy drugs/drug group	Mean dose (g/m^2^) in cases/controls	Mean years to STS	Cases/controls	Cumulative exposure to chemotherapy + adjusted for radiation dose
				OR^a^ [95% CI]
Alkylating agents category (g/m^2^)				
0		24.8	147/181	1.0 (Reference)
>0-4.99	2.3/2.6	18.6	30/28	1.4 [0.7-3.0]
5-9.99	7.2/7.1	18.4	23/19	1.6 [0.7-3.5]
10-14.99	12.1/12.4	18.9	21/12	2.3 [0.9-6.4]
≥15	43.1/35.4	16.6	25/12	2 [0.9-4.9]
Missing		23.1	29/23	
*P-value for trend*				*0.02*
Procarbazine category (g/m^2^)				
0		23.1	234/248	1.0 (Reference)
>0-6.00	2.9/4	16.2	13/8	2.2 [0.7-7.1]
>6.00	9.9/7.8	14.6	13/3	4.7 [1.3-25.1]
Missing		22.7	15/16	
*P-value for trend*				*0.02*
Alkylating agents without procarbazine category (g/m^2^)				
0		24.8	147/181	1.0 (Reference)
>0-4.99	1.6/1.9	17.9	41/37	1.6 [0.9-2.9]
5-9.99	7.4/7.3	18.3	18/13	1.9 [0.8-5.2]
10-14.99	12.2/12.5	21.4	16/11	1.6 [0.6-4.6]
≥15	47.6/35.4	16.1	21/12	1.6 [0.7-4.0]
Missing		23.1	30/21	
*P-value for trend*				*0.10*
Anthracyclines category (g/m^2^)				
0		24.5	185/208	1.0 (Reference)
>0-0.19	0.1/0.1	14.9	21/16	1.6 [0.7-4.1]
0.20-0.34	0.3/0.3	17.0	22/23	1.1 [0.5-2.5]
≥0.35	0.5/0.4	18.4	27/13	1.9 [0.8-4.4]
Missing		21.5	20/15	
*P-value for trend*				*0.16*
Platinum compounds category (g/m^2^)				
0		23.2	243/254	1.0 (Reference)
>0-0.74	0.4/0.5	14.4	12/5	1.6 [0.5-5.3]
≥0.75	2/4.1	12.6	7/5	1.2 [0.3-4.4]
Missing		21.7	13/11	
*P-value for trend*				*0.53*
Epipodophyllotoxins category (g/m^2^)				
0		23.4	234/247	1.0 (Reference)
>0-0.99	0.6/0.5	14.1	8/5	2.3 [0.6-10.8]
1-1.99	1.4/1.4	13.5	11/6	1.6 [0.5-4.9]
≥2	3.6/2.4	12.5	6/5	1.1 [0.2-5.3]
Missing		22.0	16/12	
*P-value for trend*				*0.40*
Vinca-alkaloids category (g/m^2^)				
0		26.2	150/167	1.0 (Reference)
>0-0.01	0.01/0.01	17.2	36/29	1.3 [0.7-2.8]
0.02-0.03	0.03/0.03	16.4	23/19	1.7 [0.7-4.6]
≥0.04	0.14/0.07	17.3	40/40	1.1 [0.5-2.3]
Missing		20.5	26/20	
*P-value for trend*				*0.65*
Antimetabolites category (g/m^2^)				
0		23.5	218/220	1.0 (Reference)
>0-5.99	1.9/1.1	20.4	16/12	1.6 [0.7-4.0]
6-39.99	16.8/28.2	18.2	8/12	1.7 [0.6-5.0]
≥40	91.6/77.1	15.7	10/14	0.7 [0.2-2.3]
Missing		21.0	23/17	
*P-value for trend*				*0.68*

95% CI, 95% confidence interval; OR = odds ratio.

a Conditional logistic regression matched on sex, age at childhood cancer (±1 year), calendar year of childhood cancer diagnosis (±3 years), and retinoblastoma status (heritable/non-heritable).

### Combined effects of radiotherapy dose and procarbazine exposure on subsequent STS risk

The analysis of treatment combinations highlights the joint effects of radiotherapy dose and procarbazine exposure (Table 4). At low radiation doses (<5 Gy), the risk of subsequent STS was not significantly increased in the absence of procarbazine but was statistically significant in the presence of procarbazine exposure (OR = 3.4, 95% CI 1.2-11.0). At higher radiation doses (5-19.99 Gy and ≥20 Gy), the estimated risks remained elevated irrespective of procarbazine exposure, with similar ORs across exposure categories (e.g. OR = 5.4, 95% CI 2.5-12.7 for 5-19.99 Gy alone; OR = 6.1, 95% CI 1.2-61 for the combination; OR = 24.6, 95% CI 8.7-87.8 for ≥20 Gy alone versus OR = 17.9, 95% CI 2.8-232.2 for the combination). The number of subjects in some coexposure categories was limited, resulting in wide CIs.

**Table 4:** Risk of second soft-tissue sarcoma after childhood cancer in relation to treatment combinations of radiotherapy and procarbazine

Characteristics	Cases/controls	Odds ratio^a^ [95% CI]	AR% [95% CI]	PAR% [95% CI]
Treatment combination				
0 Gy and no procarbazine	41/83	1.0 (Reference)		
0 Gy and procarbazine	5/7	2.9 [0.7 to 11.7]	65.4 [17.1 to 113.7]	1.2 [0.3 to 2.2]
>0-4.99 Gy and no procarbazine	101/127	1.5 [0.9 to 2.5]	32.7 [–2.3 to 67.6]	12.5 [–0.9 to 26.0]
>0-4.99 Gy and procarbazine	12/7	3.4 [1.2 to 11]	70.5 [37.3 to 103.7]	3.2 [1.7 to 4.7]
5-19.99 Gy and no procarbazine	39/27	5.4 [2.5 to 12.7]	81.4 [66.2 to 96.6]	12.1 [9.8 to 14.3]
5-19.99 Gy and procarbazine	6/1	6.1 [1.2 to 61]	83.6 [51.7 to 115.6]	1.9 [1.2 to 2.6]
≥20 Gy and no procarbazine	51/11	24.6 [8.7 to 87.8]	95.9 [91.3 to 100.6]	18.6 [17.7 to 19.5]
≥20 Gy and procarbazine	8/2	17.9 [2.8 to 232.2]	94.4 [82.2 to 106.6]	2.9 [2.5 to 3.2]
Missing	12/10			
*P-value for heterogeneity*		*<0.0001*		

95% CI, 95% confidence interval; AR%, attributable risk percentage; PAR%, population attributable risk percentage.

a Conditional logistic regression matched on sex, age at childhood cancer (±1 year), calendar year of childhood cancer diagnosis (±3 years), and retinoblastoma status (heritable/non-heritable).

## Discussion

### Principal findings

The results of this nested case-control study within the Pan-European cohort from 12 countries provide new evidence suggesting an increased risk of subsequent STS in tissues exposed to 5-9 Gy of radiation, alongside a clear dose–response relationship between cumulative procarbazine exposure and STS risk.

### Comparison with other studies and implications

Previous research has investigated the link between radiation dose and subsequent STS risk.^13^^,^^14^ While Menu-Branthomme et al.^13^ suggested a quadratic dose–response relationship from a nested case-control study of 23 cases of STS and of 111 controls, our findings do not indicate any departure from linearity between the cumulative radiation dose at the location of the STS and subsequent STS risk. We also observed a significantly elevated risk of subsequent STS in survivors exposed to ≥30 Gy. This finding, with greater statistical power, aligns with Jenkinson et al.,^14^ who reported a more than 50-fold [95% CI 6.0-441.5] increased risk in patients exposed to radiation doses exceeding 30 Gy compared with unexposed survivors. Prior studies reported non-significant excess risks of subsequent STS in the 5-9.9 Gy dose category of 1.5 [95% CI 0.2-9.9], 3.0 [95% CI 0.6-14.6], and 3.7 [95% CI 0.8-17.7], as reported by Menu-Branthomme et al.,^13^ Wong et al.,^28^ and Jenkinson et al.,^14^ respectively. In contrast to these prior studies that found non-significant excess risks <10 Gy, our analysis indicates a statistically significant association (OR = 3.8, 95% CI 1.3-12.0) in the 5-9 Gy range after adjustment for procarbazine exposure. This finding suggests that even relatively low radiation doses may contribute to STS risk. However, the wide CIs highlight substantial uncertainty, and differences with previous studies may reflect variations in cohort composition, sample size, or dosimetric reconstruction methods. These results therefore require confirmation in larger pooled analyses to better characterise risks at low dose levels. From a clinical perspective, these findings are particularly relevant in the context of modern radiotherapy techniques such as intensity-modulated radiotherapy, which reduce high-dose exposure but may increase the volume of normal tissue receiving low doses. While the absolute risk at low doses remains modest, our results support efforts to minimise unnecessary radiation exposure wherever feasible. In survivorship care, adherence to established follow-up guidelines remains essential, with particular attention to high-risk groups, including patients treated for neuroblastoma or bone sarcoma and those exposed to radiation in breast or pelvic regions.

Our data suggest an increased risk of subsequent STS among childhood cancer survivors treated with procarbazine, consistent with one previous finding that had reported a specifically elevated risk of subsequent STS (OR = 6.7, 95% CI 1.1-56.9) compared with those who did not receive procarbazine.^13^ However, a previous study did not demonstrate a dose–response relationship for procarbazine, likely due to low statistical power.^13^ Therefore, our large case-control study has reported the first linear dose–effect relationship between the cumulative dose of procarbazine and subsequent STS risk, improving the understanding of procarbazine’s carcinogenicity. Although the International Agency for Research on Cancer has classified procarbazine as carcinogenic in the context of combination therapy, the independent contribution of this agent remains difficult to disentangle.^29^ In this study, we observed that 95% (46/48) and 31% (15/48) of patients treated with procarbazine also received vinca-alkaloids and anthracyclines, respectively. No effects were identified for other alkylating agents, platinum compounds, epipodophyllotoxins, vinca-alkaloids, anthracyclines, and antimetabolites. Contrary to the findings of Henderson et al.,^15^ we observed no evidence of an association between cumulative anthracycline dose and subsequent STS risk (Supplementary Table S9, available at https://doi.org/10.1016/j.esmoop.2026.108313). Discrepancies between studies may be partly explained by methodological differences, such as failing to distinguish between sarcoma types (our study only considered STS), the small number of STS cases in the Henderson et al. study (<25% of those the current study), and a considerably shorter median follow-up in the Henderson study. Although a previous study has indicated that anthracycline-based chemotherapy is associated with an increased risk of breast cancer in female childhood cancer survivors,^30^ our results suggest that anthracyclines were not an independent risk factor for subsequent STS even when focusing solely on female survivors (data not shown). We also found that 75% (36/48) of patients treated with procarbazine also received radiotherapy. Similar to findings in some studies,^14^ we identified the highest risk of subsequent STS among survivors who underwent both radiotherapy and procarbazine treatment (Supplementary Table S6, available at https://doi.org/10.1016/j.esmoop.2026.108313, Table 4). Notably, even at low radiation doses (<5 Gy), patients exposed to procarbazine had a significant risk of subsequent STS. This underscores the need for further research into their combined effects. The precise biological mechanisms underlying the interaction between radiation and procarbazine in carcinogenesis are not fully understood. However, *in vitro* studies suggest several plausible mechanisms for their synergistic effects, including disruptions in normal DNA repair and cell cycle progression, thereby enhancing carcinogenesis.^31^^,^^32^

### Limitations

Several limitations should be considered when interpreting our findings. Firstly, dose reconstruction relied on historical treatment records and voxel-based phantoms, and some degree of radiation dose uncertainties and misclassification are unavoidable, particularly for older treatments. Such misclassification is likely non-differential and would tend to attenuate observed associations. Secondly, missing data on radiation dose at the site of the subsequent STS (*n* = 14) and detailed chemotherapy data (*n* = 12) were limited and unlikely to materially affect significantly the results. Thirdly, AR and PAR estimates should be interpreted cautiously, as they rely on untestable assumptions regarding causality inherent to AR calculations in observational studies. Accordingly, these estimates should be viewed as model-based indicators of the potential public health relevance of an exposure under these assumptions, rather than as definitive causal quantities.^25^ Our analysis focused on therapeutic exposures from 1944 to 2001, reflecting treatment regimens over several decades. Although procarbazine is no longer administered in most contemporary cancer treatment protocols, it was widely used for several decades from the 1960s onwards. Therefore, our findings are crucial for updating evidence-based clinical follow-up guidelines regarding subsequent STS risk. Given the ongoing use of external beam radiation in current treatment approaches, our results regarding radiation-related risks are particularly relevant. We focused on subsequent STS diagnosed at least 5 years after FPN, which may have caused us to miss risks for earlier tumours. Importantly, despite our nested case-control design, which included matching on subcohort, sex, age, and calendar year of the FPN, as well as hereditary retinoblastoma status, and extensive adjustment for treatment-related factors, residual confounding cannot be entirely excluded. Confounding by indication may persist, as treatment decisions were influenced by disease severity, treatment era, and clinical judgement. Furthermore, the intrinsic correlation between radiotherapy and chemotherapy in multimodal treatment complicates the disentanglement of their independent effects. Our analysis of combined exposures suggests potential interaction patterns (Table 4), although limited statistical power in some subgroups warrants cautious interpretation. In addition, data on several potential confounders were unavailable, including environmental exposures and genetic predisposition syndromes such as Li-Fraumeni syndrome or neurofibromatosis type 1. Although hereditary retinoblastoma was accounted for through matching and sensitivity analyses, the influence of other genetic susceptibility factors cannot be excluded (Supplementary Table S5, available at https://doi.org/10.1016/j.esmoop.2026.108313). Overall, our findings should therefore be interpreted as strong and consistent associations, rather than definitive evidence of causality. The evaluation of multiple subgroup and interaction analyses may have increased the risk of type I error and false-positive findings. Moreover, some analyses may have been underpowered, limiting the ability to detect true effect modification. Results relating to high-risk subgroups should therefore be interpreted with caution, particularly given the exploratory nature of certain analyses and the absence of formal correction for multiple comparisons. In addition, subgroup-specific estimates represent marginal effects and may not fully capture the combined influence of multiple patient- or treatment-related characteristics. As such, these findings should be considered hypothesis-generating rather than conclusive. Greater emphasis should be placed on the consistency of observed patterns, effect estimates, and their clinical and biological plausibility. Future studies with larger sample sizes and adequate statistical power will be required to confirm these findings. Finally, the generalisability of the study may be limited by its European cohort, which may not fully represent childhood cancer survivors from other regions with different health care systems and treatment practices. Nevertheless, the nested case-control design within the well-characterized PanCareSurFup cohort,^9^^,^^17^, ^18^, ^19^ which spans multiple countries and encompasses diverse populations, enhances the validity and reliability of our findings.

### Conclusion

Although high radiation doses remain the primary risk factor for secondary STS, our findings suggest a possible increase in risk at lower doses (5-9 Gy) and following procarbazine treatment among childhood cancer survivors. These observations warrant further investigation and may merit consideration in treatment planning and long-term follow-up guidelines for cancer survivors.
