# Supplementary material for: Cancer treatments-related factors for subsequent soft-tissue sarcoma in childhood cancer survivors
Source: ESMO Open. 2026 Jul 24;11(8):108313. doi: 10.1016/j.esmoop.2026.108313 (PMC13449366; doi:10.1016/j.esmoop.2026.108313)
Supplement: Supplementary file 1 [file mmc1.docx]

Supplemental materials

**Supplementary Table S1**: Frequency and cumulative dose of cytotoxic agents in second soft-tissue sarcoma cases and their controls among childhood cancer survivors

| **Classes of cytotoxics** | **Cytotoxics name** | **Cases (n = 275)**  **n exposed; *Mean [min-max] (g/m^2^)*** | **Controls (n = 275)**  **n exposed; *Mean [min-max] (g/m^2^)*** |
| --- | --- | --- | --- |
| Alkylating agents | Cyclophosphamide | n=64; *11.38[0.02-171.55*] | n=54; *8.94[0-30.41]* |
|  | Procarbazine | n=31; *6.39[0.09-18.01]* | n=17; *5.06[0.08-8.51]* |
|  | Ifosfamide | n=21; *32.81[0.13-115.25]* | n=9; *24.99[1.73-84]* |
|  | Mustine | n=15; *0.05[0.01-0.1]* | n=4; *0.04[0.01-0.07]* |
|  | Chlorambucil | n=7; *0.4[0.23-0.76]* | n=6; *0.56[0.45-0.73]* |
|  | Melphalan | n=6; *0.24[0.04-0.65]* | n=1; *0.15[0.15-0.15]* |
| Anthracyclines | Adriamycin | n=60; *0.29[0.02-0.9]* | n=42; *0.26[0.01-0.5]* |
|  | Daunorubicin | n=6; *0.25[0.13-0.45]* | n=14; *0.17[0.05-0.5]* |
|  | Epirubicin | n=5; *0.32[0.1-0.56]* | n=2; *0.33[0.3-0.35]* |
| Vinka-alkaloids | Vincristine | n=93; *0.06[0-2.56]* | n=80; *0.03[0-0.18]* |
|  | Vinblastine | n=14; *0.04[0-0.15]* | n=12; *0.06[0.01-0.16]* |
|  | Vindesine | n=4; *0.01[0-0.01]* | n=5; *0.01[0-0.02]* |
| Epipodophyllotoxins | VP-16 (Etoposide) | n=16; *0.41[0.12-1.13]* | n=7; *0.46[0.08-0.72]* |
|  | VM-26 (Teniposide) | n=8; *1.6[0.6-2.8]* | n=5; *3.94[0.6-12]* |
| Platinum compounds | Cisplatin | n=15; *2.07[0.08-6.83]* | n=12; *1.78[0.06-6.36]* |
|  | Carboplatin | n=21; *3.73[0.04-13.52]* | n=22; *5.11[0.04-25.76]* |
| Glucocorticoids | Prednisone | n=5; *0.16[0.03-0.3]* | n=3; *0.37[0.02-0.61]* |
|  | Prednisolone | n=16; *8.67[0.2-89.88]* | n=18; *8.95[0.08-37.23]* |
|  | Dexamethasone | n=12; *44.41[0.23-90.94]* | n=24; *31.21[0.05-53.18]* |
| Anti-metabolites | Cytarabine | n=4; *1.27[0.47-2.25]* | n=6; *1.19[0.09-1.84]* |
|  | Mercaptopurine | n=64; *11.38[0.02-171.55]* | n=54; *8.94[0-30.41]* |
|  | Thioguanine | n=31; *6.39[0.09-18.01]* | n=17; *5.06[0.08-8.51]* |

Abbreviations: min = minimum; max = Maximum.

**Supplementary Table S2**: Characteristics of childhood cancers survivors who developed a subsequent Soft-tissue sarcoma (STS) according the first primary neoplasm (FPN)

|  | **Leukemia**  **No. (%)** | **Hodgkin lymphoma**  **No. (%)** | **Non-Hodgkin lymphoma**  **No. (%)** | **Central Nervous System**  **No. (%)** | **Neuroblastoma**  **No. (%)** | **Retinoblastoma**  **No. (%)** | **Wilms tumor**  **No. (%)** | **Bone sarcoma**  **No. (%)** | **Soft-tissue sarcoma^π^**  **No. (%)** | **Other and not classifiable**  **No. (%)** |
| --- | --- | --- | --- | --- | --- | --- | --- | --- | --- | --- |
| **Overall n (%)** | **25 (9.1)** | **31 (11.3)** | **7 (2.5)** | **39 (14.2)** | **16 (5.8)** | **56 (20.4)** | **31 (11.3)** | **21 (7.6)** | **20 (7.3)** | **29 (10.5)** |
| **Age at Diagnosis of FPN (years)** |  |  |  |  |  |  |  |  |  |  |
| *Median (min-max)* | 4 (0.9-13.5) | 13.5 (3.8-18.7) | 14.9 (5.8-17.7) | 8.3 (0.9-19.2) | 1 (0.2-3.9) | 0.8 (0.1-3.7) | 4.5 (0.9-11.7) | 12.9 (5.3-19.1) | 3.7 (0.4-15.3) | 13.7 (0.7-19.7) |
| <1 | 1 (4.0) |  |  | 1 (2.6) | 8 (50.0) | 32 (57.1) | 1 (3.2) | - | 2 (10.0) | 2 (6.9) |
| 0–3 | 11 (44.0) | 1 (3.2) | - | 7 (17.9) | 8 (50.0) | 24 (42.9) | 13 (41.9) | - | 8 (40.0) | 4 (13.8) |
| 4–7 | 10 (40.0) | 3 (9.7) | 2 (28.6) | 8 (20.5) | - | - | 14 (45.2) | 1 (4.8) | 3 (15.0) | 3 (10.3) |
| 8–11 | 2 (8.0) | 6 (19.4) | 1 (14.3) | 11 (28.2) | - | - | 3 (9.7) | 5 (23.8) | 4 (20.0) | 3 (10.3) |
| 12–20 | 1 (4.0) | 21 (67.7) | 4 (57.1) | 12 (30.8) | - | - | - | 15 (71.4) | 3 (15.0) | 17 (58.6) |
| **Decade of Diagnosis of FPN** |  |  |  |  |  |  |  |  |  |  |
| *Median (min-max)* | 1984  (1967-2001) | 1979  (1959-1997) | 1976  (1955-1988) | 1979  (1944-1998) | 1971  (1952-2001) | 1961  (1944-1990) | 1975  (1950-1994) | 1978  (1963-2001) | 1976  (1957-1999) | 1975  (1958-1996) |
| <1970 | 2 (8.0) | 4 (12.9) | 3 (42.9) | 15 (38.5) | 6 (37.5) | 43 (76.8) | 10 (32.3) | 3 (14.3) | 6 (30.0) | 8 (27.6) |
| 1970-1979 | 6 (24.0) | 12 (38.7) | 3 (42.9) | 5 (12.8) | 6 (37.5) | 2 (3.6) | 10 (32.3) | 9 (42.9) | 8 (40.0) | 11 (37.9) |
| 1980-1989 | 10 (40.0) | 10 (32.3) | 1 (14.3) | 15 (38.5) | 2 (12.5) | 10 (17.9) | 9 (29.0) | 6 (28.6) | 3 (15.0) | 7 (24.1) |
| ≥ 1990 | 7 (28.0) | 5 (16.1) | - | 4 (10.3) | 2 (12.5) | 1 (1.8) | 2 (6.5) | 3 (14.3) | 3 (15.0) | 3 (10.3) |
| **Attained Age** **at subsequent STS**^¥^ **(years)** |  |  |  |  |  |  |  |  |  |  |
| *Median (min-max)* | 22.2 (9.8-40.1) | 29.7 (10.5-56) | 33.2 (16.2-58.8) | 27.7 (11.4-57) | 23.5 (9.4-43.5) | 37 (6-62.1) | 25.5 (7.6-47.3) | 29.4 (19.3-52.5) | 21.9 (10.1-61.3) | 28 (12.9-60.8) |
| 5–14 | 5 (20.0) | 2 (6.5) | - | 2 (5.1) | 2 (12.5) | 6 (10.7) | 6 (19.4) | - | 5 (25.0) | 3 (10.3) |
| 15–24 | 11 (44.0) | 8 (25.8) | 2 (28.6) | 15 (38.5) | 7 (43.8) | 5 (8.9) | 9 (29.0) | 6 (28.6) | 6 (30.0) | 6 (20.7) |
| 25–34 | 8 (32.0) | 13 (41.9) | 3 (42.9) | 12 (30.8) | 4 (25.0) | 12 (21.4) | 11 (35.5) | 8 (38.1) | 4 (20.0) | 13 (44.8) |
| 35–44 | 1 (4.0) | 6 (19.4) | - | 8 (20.5) | 3 (18.8) | 17 (30.4) | 4 (12.9) | 5 (23.8) | 2 (10.0) | 4 (13.8) |
| ≥ 45 | - | 2 (6.5) | 2 (28.6) | 2 (5.1) | - | 16 (28.6) | 1 (3.2) | 2 (9.5) | 3 (15.0) | 3 (10.3) |
| **Years from FPN Diagnosis**^≠^ |  |  |  |  |  |  |  |  |  |  |
| *Median (min-max)* | 17 (7.2-32.6) | 13 (5.2-39.4) | 15.5 (6.6-43.9) | 17.5 (5.9-45.4) | 22.8 (8.3-40.9) | 35.9 (5.7-61.6) | 20.7 (5.6-42.5) | 18 (7.7-39.6) | 16 (6.4-50) | 15.1 (6.2-41.6) |
| 5–9 | 5 (20.0) | 10 (32.3) | 2 (28.6) | 4 (10.3) | 1 (6.3) | 2 (3.6) | 6 (19.4) | 5 (23.8) | 5 (25.0) | 5 (17.2) |
| 10–19 | 11 (44.0) | 10 (32.3) | 2 (28.6) | 18 (46.2) | 4 (25.0) | 8 (14.3) | 7 (22.6) | 10 (47.6) | 8 (40.0) | 13 (44.8) |
| 20–29 | 7 (28.0) | 8 (25.8) | 1 (14.3) | 12 (30.8) | 7 (43.8) | 4 (7.1) | 12 (38.7) | 4 (19.0) | 3 (15.0) | 9 (31) |
| 30–39 | 2 (8.0) | 3 (9.7) | 1 (14.3) | 3 (7.7) | 3 (18.8) | 21 (37.5) | 4 (12.9) | 2 (9.5) | 2 (10.0) | - |
| ≥ 40 | - | - | 1 (14.3) | 2 (5.1) | 1 (6.3) | 21 (37.5) | 2 (6.5) | - | 2 (10.0) | 2 (6.9) |
| **Deaths n (%)** | 10 (40.0) | 22 (71.0) | 5 (71.4) | 32 (82.1) | 11 (68.8) | 32 (57.1) | 22 (71.0) | 10 (47.6) | 14 (70.0) | 15 (51.7) |

*No. = frequency; FPN =* *first primary neoplasm;* ^¥^*STS = soft-tissue sarcoma.* ^≠^*Years from FPN Diagnosis = Years between FPN diagnosis and STS diagnosis. ^π^Survivors who developed a first and a second soft tissue sarcomas.*

**Supplementary Table S3**: Radiation dose–response relationship for risks of second soft-tissue sarcoma (STS) - Excess odds ratio (EOR) models of subsequent STS per Gy of dose of radiation received to site of STS

| **Exposures in model** | **Cumulative radiation dose** | **P-values from LRT** | **Cumulative radiation dose + adjusted for Procarbazine** | **P-values from LRT** |
| --- | --- | --- | --- | --- |
|  | **EOR/Gy [95% CI]** |  | **EOR/Gy [95% CI]** |  |
| Null model | — | *—* | + Procarbazine | *—* |
| Radiation, Gy* | 0.86 [0.35-2.16] | ***<0.001****^a^* | 0.83 [0.34-2.04] | ***0.0162****^a^* |
| Radiation* + radiation quadratic | 0.92 [0.29-2.35] | *0.798^b^* | 0.89 [0.25-2.37] | *0.107^b^* |
| Radiation* + radiation quadratic + radiation cubic | 1.18 [0.32-3.45] | *0.410^c^* | 1.11 [0.24-3.28] | *0.520^c^* |

EOR/Gy: Excess odds ratio of subsequent STS per Gy of dose of radiation received to site of STS; 95% CI = 95% confidence interval generally estimated using the profile likelihood, and when the profile CI did not converge were Wald-based. *Fitted as a linear trend continuous in dose. LRT= likelihooRed-ratio test; ^a^P-value relative to the null model or null model + Procarbazine; ^b^P-value related to likelihood-ratio test resulting from extending the existing model (Linear EOR model) by the additional term radiation quadratic (in linear part) and ^c^P-values related to likelihood-ratio test resulting from linear-quadratic extended to linear-quadratic-cubic. The univariable linear-quadratic model yielded an EOR/Gy of 0.92 [95% CI: 0.29-2.35] and an EOR/Gy^2^ of -0.003 [95% CI: -0.027-0.040], while the univariable linear–quadratic–cubic model yielded an EOR/Gy of 1.18 [95% CI: 0.32-3.45], an EOR/Gy^2^ of -0.037 [95% CI: -0.16-0.05] and an EOR/Gy^3^ of 0.0007 [95% CI: -0.001-0.003].

**Supplementary Table S4**: Evaluation of potential effect modifiers of the linear radiation dose-response for subsequent STS among childhood cancer survivors (PanCareSurFup)

| **Characteristics** | **Cumulative radiation dose : mean, in Gy^ψ^** | | **Cases / Controls** | **EOR/Gy [95% CI] of cumulative radiation dose** |
| --- | --- | --- | --- | --- |
|  | **Cases** | **Controls** |  |  |
| **Sex** |  |  |  |  |
| Female | 13.0 (0-65.7) | 3.8 (0-43.3) | 97/76 | 2.35 [0.65-8.14] |
| Male | 13.3 (0-58.9) | 5.5 (0-59.1) | 125/101 | 0.33 [0.12-0.93] |
| *P_interaction_* |  |  |  | ***0.02*** |
| **First Primary Neoplasm (FPN) diagnosis** |  |  |  |  |
| Leukemia | 7.9 (0-21) | 3.6 (0-20.8) | 22/25 | 0.58 [0.08-2.96] |
| Hodgkin lymphoma | 16.3 (0-42.1) | 8.1 (0-32.8) | 25/16 | 0.69 [0.13-3.35] |
| Non-Hodgkin lymphoma | 16.5 (0.2-34.7) | 6.9 (0-33.8) | 06/10 | 0.07 [-0.03-1.10] |
| Central Nervous System | 10.0 (0-58.9) | 3.1 (0-15.2) | 29/23 | 0.67 [0.08-5.58] |
| Neuroblastoma | 14.6 (1.1-28) | 9.4 (0.1-28.2) | 16/5 | 5.52 [0.71-52.31] |
| Retinoblastoma | 3.9 (0-41.2) | 3.4 (0-43.3) | 49/41 | 0.05 [-0.02-1.01] |
| Wilms tumor | 24.7 (0.1-52.5) | 11.9 (0.1-59.1) | 28/20 | 1.17 [0.30-4.73] |
| Bone sarcoma | 21.2 (0-58.7) | 0.4 (0-1.1) | 17/7 | 4.16 [0.40-24.72] |
| Soft-tissue sarcoma | 15.8 (0-65.7) | 2 (0-29.9) | 13/19 | 0.96 [0.09-5.07] |
| Other and not classifiable | 17.5 (0.3-53.2) | 1.8 (0-13.8) | 17/11 | 4.17 [0.72-23.29] |
| *P_interaction_* |  |  |  | ***0.02*** |
| **Age at Diagnosis of FPN (years)** |  |  |  |  |
| < 2 year old | 9.1 (0-49.2) | 5.3 (0-59.1) | 76/59 | 0.63 [0.13-3.22] |
| 2–10 years old | 13.8 (0-65.7) | 5.5 (0-33.8) | 76/65 | 0.89 [0.25-3.07] |
| 10–20 years old | 17.3 (0-58.9) | 3.3 (0-32.8) | 70/53 | 0.64 [0.19-2.22] |
| *P_interaction_* |  |  |  | *0.47* |
| **Decade of Diagnosis of FPN** |  |  |  |  |
| < 1970 | 7.3 (0-52.5) | 4.1 (0-59.1) | 85/64 | 2.01 [0.27-14.20] |
| 1970-1980 | 18.2 (0-65.7) | 5 (0-33.8) | 63/54 | 0.58 [0.17-2.12] |
| 1980-1990 | 13.2 (0-55.8) | 4.2 (0-43.3) | 54/46 | 1.05 [0.21-4.89] |
| ≥ 1990 | 20.6 (0.1-58.9) | 9.3 (0-32.8) | 20/13 | 0.30 [0.04-1.67] |
| *P_interaction_* |  |  |  | *0.39* |
| **Attained Age** **(years)** |  |  |  |  |
| < 20 | 16.6 (0-65.7) | 7.7 (0-43.3) | 50/38 | 0.81 [0.15-5.59] |
| ≥ 20-35 | 14.9 (0-58.9) | 4.7 (0-59.1) | 106/90 | 0.61 [0.21-1.82] |
| ≥ 35 | 7.5 (0-52.5) | 2.7 (0-28.2) | 66/49 | 0.97 [0.15-5.36] |
| *P_interaction_* |  |  |  | ***0.01*** |
| **Years from FPN Diagnosis (years)**^≠^ |  |  |  |  |
| < 15 | 18.5 (0-65.7) | 7.2 (0-43.3) | 71/57 | 0.46 [0.13-1.59] |
| ≥ 15-25 | 13.2 (0-58.7) | 4.1 (0-59.1) | 67/49 | 2.96 [0.48-14.43] |
| ≥ 25 | 8.4 (0-52.5) | 3.3 (0-34) | 84/71 | 0.52 [0.10-2.42] |
| *P_interaction_* |  |  |  | *0.09* |
| **Subsequent STS**^¥^ **by histological type** |  |  |  |  |
| Fibromatous neoplasms | 16.5 (0-65.7) | 5.7 (0-29.9) | 32/23 | 1.64 [0.17-11.51] |
| Lipomatous neoplasms | 11.3 (0.1-49.4) | 3.8 (0-28.2) | 17/17 | 0.78 [0.02-11.41] |
| Myomatous neoplasms | 7.9 (0-55.8) | 3.2 (0-43.3) | 75/57 | 0.94 [0.13-5.62] |
| Nerve sheath tumors | 14.7 (0-42.1) | 4.2 (0-32.8) | 35/27 | 0.57 [0.09-4.90] |
| Soft-tissue tumors and sarcomas, NOS | 11.2 (0-53.2) | 2.8 (0-14.2) | 20/21 | 0.23 [0.01-1.66] |
| Others STS^¥^ | 20.3 (0-58.9) | 9.5 (0-59.1) | 43/32 | 1.03 [0.18-7.32] |
| *P_interaction_* |  |  |  | *0.14* |
| **Subsequent STS**^¥^ **location** |  |  |  |  |
| Breast, uterine and pelvic area | 11.7 (0-58.7) | 1.7 (0-34) | 55/46 | 2.16 [0.39-9.08] |
| Other parts of the body | 13.7 (0-65.7) | 5.9 (0-59.1) | 167/131 | 0.52 [0.21-1.33] |
| *P_interaction_* |  |  |  | *0.14* |
| **Chemotherapy (CT)** |  |  |  |  |
| No | 9.6 (0-58.9) | 3.9 (0-59.1) | 82/77 | 0.39 [0.11-1.22] |
| Yes | 15.1 (0-65.7) | 5.5 (0-34) | 140/100 | 0.97 [0.39-2.37] |
| *P_interaction_* |  |  |  | *0.10* |
| **Alkylating agent** |  |  |  |  |
| No | 11.6 (0-58.9) | 4.8 (0-59.1) | 119/112 | 054 [0.19-1.52] |
| Yes | 15.9 (0-65.7) | 5.3 (0-34) | 83/54 | 1.08 [0.37-3.10] |
| Missing | 10.8 (0-40.7) | 2.1 (0-8.8) | 20/11 |  |
| *P_interaction_* |  |  |  | *0.08* |
| **Procarbazine** |  |  |  |  |
| No | 13 (0-65.7) | 4.7 (0-59.1) | 191/165 | 0.71 [0.29-1.79] |
| Yes | 12.4 (0-42.1) | 7.3 (0-30.1) | 26/10 | 1.40 [0.26-6.25] |
| Missing | 28.4 (0-40.7) | 0.2 (0.1-0.3) | 5/2 |  |
| *P_interaction_* |  |  |  | *0.13* |
| **Anthracyclines** |  |  |  |  |
| No | 10.1 (0-58.9) | 4.5 (0-59.1) | 156/130 | 0.66 [0.24-1.77] |
| Yes | 19.8 (0-65.7) | 6 (0-32.8) | 61/45 | 1.01 [0.36-2.81] |
| Missing | 28.4 (0-40.7) | 0.2 (0.1-0.3) | 5/2 |  |
| *P_interaction_* |  |  |  | *0.66* |

Abbreviations: FPN= first primary neoplasm; ^≠^Years from FPN Diagnosis= Years between FPN diagnosis and STS diagnosis; ^¥^STS= soft-tissue sarcoma [Kaposi’s sarcoma (n=8), synovial-like neoplasms (n=8), miscellaneous bone tumors (n=4), complex mixed and stromal neoplasms (n=3), blood vessel tumors (n=2), neurilemoma malignant (n=2), miscellaneous tumors (n=2), and others]; ^ψ^mean (minimum - Maximum) of the reconstructed/estimated dose to the site of the STS and an equivalent dose among control subjects. See Methods section for details on dose reconstruction. EOR/Gy: Excess odds ratio of subsequent STS per Gy of dose of radiation received to site of STS; 95% CI = 95% confidence interval generally estimated using the profile likelihood, and when the profile CI did not converge were Wald-based. *P_interaction_* from the likelihood ratio statistic test between model containing the linear trend in continuous cumulative radiation dose and categorical covariate in a loglinear term, and with that with the categorical covariate in an interaction with cumulative radiation dose.

**Supplementary Table S5**: Risk of subsequent soft-tissue sarcoma in relation to cumulative exposure to specific types of cytotoxics crude and adjusted for dose of radiation received to site of soft-tissue sarcoma excluding retinoblastoma survivors.

| **Classes of cytotoxics** | **Mean dose (g/m^2^) in Cases / Controls** | **Mean years to STS** | **Cases / Controls** | **+ adjusted for radiation dose** |
| --- | --- | --- | --- | --- |
|  |  |  |  | **Odds ratio^$^ [95% CI]** |
| **Alkylating agents category** |  |  |  |  |
| 0 g/m^2^ |  | 22.2 | 109/136 | **1.0 (Reference)** |
| >0-4.99 | 2.47/2.72 | 17.2 | 26/26 | 1.4 [0.6-3.1] |
| 5-9.99 | 7.31/7.22 | 18.0 | 20/17 | 1.6 [0.7-4.0] |
| 10-14.99 | 11.84/12.27 | 15.0 | 18/11 | 1.8 [0.7-5.5] |
| >=15 | 37.94/35.89 | 15.0 | 23/11 | 1.9 [0.8-5.0] |
| Missing |  | 18.7 | 23/18 |  |
| *P-value for trend* |  |  |  | *0.07* |
| **Procarbazine category** |  |  |  |  |
| 0 g/m^2^ |  | 20.1 | 178/195 | **1.0 (Reference)** |
| >0-6.00 | 2.9/4.03 | 14.8 | 13/8 | 2.2 [0.7-7.6] |
| >6.00 | 9.87/7.79 | 15.2 | 13/3 | 4.8 [1.3-26.2] |
| Missing |  | 20.0 | 15/13 |  |
| *P-value for trend* |  |  |  | ***0.02*** |
| **Alkylating agents without procarbazine category** |  |  |  |  |
| 0 g/m^2^ |  | 22.2 | 109/136 | **1.0 (Reference)** |
| >0-4.99 | 1.69/1.95 | 16.5 | 39/35 | 1.6 [0.8-3.0] |
| 5-9.99 | 7.66/7.4 | 16.8 | 15/11 | 2.2 [0.7-7.1] |
| 10-14.99 | 11.85/12.43 | 19.3 | 13/10 | 1.0 [0.3-3.4] |
| >=15 | 41.74/35.89 | 13.8 | 19/11 | 1.6 [0.6-4.3] |
| Missing |  | 18.4 | 24/16 |  |
| *P-value for trend* |  |  |  | *0.25* |
| **Anthracyclines category** |  |  |  |  |
| 0 g/m^2^ |  | 21.1 | 132/155 | **1.0 (Reference)** |
| >0-0.19 | 0.11/0.1 | 13.9 | 21/16 | 1.5 [0.6-3.9] |
| 0.20-0.34 | 0.27/0.28 | 17.9 | 20/23 | 0.9 [0.4-2.2] |
| >=0.35 | 0.5/0.44 | 17.8 | 26/13 | 1.7 [0.8-4.2] |
| Missing |  | 18.3 | 20/12 |  |
| *P-value for trend* |  |  |  | *0.29* |
| **Platinum compounds category** |  |  |  |  |
| 0 g/m^2^ |  | 20.3 | 189/203 | **1.0 (Reference)** |
| >0-0.74 | 0.35/0.52 | 12.1 | 11/5 | 1.4 [0.5-4.9] |
| >=0.75 | 1.97/2.32 | 13.5 | 6/3 | 1.6 [0.4-7.2] |
| Missing |  | 16.7 | 13/8 |  |
| *P-value for trend* |  |  |  | *0.42* |
| **Epipodophyllotoxins category** |  |  |  |  |
| 0 g/m^2^ |  | 20.4 | 179/196 | **1.0 (Reference)** |
| >0-0.99 | 0.58/0.47 | 14.6 | 8/5 | 2.4 [0.6-11.7] |
| 1-1.99 | 1.42/1.43 | 12.5 | 10/4 | 1.9 [0.6-7.4] |
| >=2 | 3.6/2.37 | 12.6 | 6/5 | 1.1 [0.2-5.7] |
| Missing |  | 18.6 | 16/9 |  |
| *P-value for trend* |  |  |  | *0.30* |
| **Vinca-alkaloids category** |  |  |  |  |
| 0 mg/m^2^ |  | 22.3 | 98/116 | **1.0 (Reference)** |
| >0-0.01 | 0.01/0.01 | 17.8 | 33/27 | 1.4 [0.7-3.0] |
| 0.02-0.03 | 0.03/0.03 | 15.2 | 23/19 | 1.8 [0.7-4.8] |
| >=0.04 | 0.14/0.07 | 17.2 | 40/40 | 1.0 [0.5-2.3] |
| Missing |  | 18.4 | 25/17 |  |
| *P-value for trend* |  |  |  | *0.71* |
| **Antimetabolites category** |  |  |  |  |
| 0 mg/m^2^ |  | 20.3 | 165/167 | **1.0 (Reference)** |
| >0-5.99 | 1.99/1.1 | 16.3 | 14/12 | 1.4 [0.6-3.6] |
| 6-39.99 | 17.6/28.16 | 18.0 | 7/12 | 1.6 [0.5-5.0] |
| >=40 | 91.62/77.08 | 16.1 | 10/14 | 0.6 [0.2-2.2] |
| Missing |  | 17.5 | 23/14 |  |
| *P-value for trend* |  |  |  | *0.89* |

Abbreviations: 95% CI = 95% confidence interval. **^$^**Conditional logistic regression matched on sex, age at childhood cancer (± 1 year), calendar year of childhood cancer diagnosis (± 3 years), and retinoblastoma status (heritable/non-heritable).

#### Supplementary Table S6: General and treatment characteristics of childhood cancer survivors treated with procarbazine

| **Characteristics** | **Patients treated with procarbazine (n = 48)** | **Patients treated with procarbazine at doses ≥6.0 g/m2 (n = 16)** |
| --- | --- | --- |
| **Sex** |  |  |
| Female | 17 (35.4) | 7 (43.8) |
| Male | 31 (64.6) | 9 (56.3) |
| **First Primary Neoplasm (FPN) Diagnosis** |  |  |
| Leukemia | 1 (2.1) | - |
| Hodgkin lymphoma | 36 (75) | 11 (68.8) |
| Non-Hodgkin lymphoma | 2 (4.2) | 1 (6.3) |
| Central Nervous System | 2 (4.2) | 1 (6.3) |
| Bone sarcoma | 3 (6.3) | 2 (12.5) |
| Soft-tissue sarcoma | 1 (2.1) | - |
| Other and not classifiable | 3 (6.3) | 1 (6.3) |
| **Age at Diagnosis of FPN (years)** |  |  |
| *Median (min-max)* | *12 (2.9-19.1)* | *12 (3.8-18.6)* |
| 1–3 | 4 (8.3) | 1 (6.3) |
| 4–7 | 5 (10.4) | 2 (12.5) |
| 8–11 | 12 (25) | 3 (18.8) |
| 12–20 | 27 (56.3) | 10 (62.5) |
| **Decade of Diagnosis of FPN** |  |  |
| *Median (min-max)* | *1979 (1967-1997)* | *1979 (1967-1986)* |
| <1970 | 3 (6.3) | 1 (6.3) |
| 1970-1979 | 21 (43.8) | 9 (56.3) |
| 1980-1989 | 20 (41.7) | 6 (37.5) |
| ≥ 1990 | 4 (8.3) | - |
| **Radiotherapy (RT)** |  |  |
| No | 12 (25) | 3 (18.8) |
| Yes | 36 (75) | 13 (81.3) |

**Supplementary Table S7**: Cumulative exposure to specific types of chemotherapy is related to the excess odds ratio (EOR) of second soft-tissue sarcoma (STS) adjusted for radiation dose received to site of STS

| **Specific types of chemotherapy** | **Drugs/drug groups** | **EOR/g/m^2^ [95% CI]** | **P-values from LRT** |
| --- | --- | --- | --- |
| **Alkylating agents** | Null model with radiation | — | *—* |
|  | + Alkylating agents (g/m^2^) (AA) | 0.02 [-0.01-0.15] | 0.31^a^ |
|  | + AA (CED – cyclophosphamide equivalent dose) | 0.05 [-0.01-0.19] | 0.18^a^ |
|  | + AA without Procarbazine (g/m^2^) | 0.01 [-0.01-0.08] | 0.40^a^ |
| **Specific alkylating agents** | *+ Cyclophosphamide (g/m^2^)* | *-0.004 [-0.03-0.08]* | *0.87^a^* |
|  | *+ Procarbazine (g/m^2^)* | ***0.46 [0.01-2.66]*** | ***<0.001****^a^* |
|  | *+ Ifosfamide (g/m^2^)* | *0.009 [-0.01-0.14]* | *0.60^a^* |
| **Other cytotoxic groups or specific cytotoxics** | + Anthracyclines (g/m^2^) | 1.50 [-0.61-6.77] | 0.24^a^ |
|  | *+ Adriamycin (g/m^2^)* | *0.62 [-1.49-6.11]* | *0.68^a^* |
|  | + Platinum compounds (g/m^2^) | -0.07 [**-1.92] | 0.85^a^ |
|  | *+ Carboplatin (g/m^2^)* | *-0.08 [**-2.36]* | *0.86^a^* |
|  | + Epipodophyllotoxins (g/m^2^) | 0.70 [-0.26-4.35] | 0.26^a^ |
|  | + Vinca-alkaloids (g/m^2^) | 0.64 [-0.36-10.58] | 0.52^a^ |
|  | *+ Vincristine (g/m^2^)* | *0.68 [-0.36-11.82]* | *0.51^a^* |
|  | + Antimetabolites (g/m^2^) | -0.005 [**-0.02] | 0.19^a^ |
|  | *+ Mercaptopurine (g/m^2^)* | *-0.006 [-0.02-0.04]* | *0.68^a^* |
|  | *+ Thioguanine (g/m^2^)* | *-0.11 [-0.43-3.16]* | *0.82^a^* |

EOR/g/m^2^: Excess odds ratio of subsequent STS per g/m^2^ of cumulative exposure to specific types of chemotherapy classes (in grey) or individual drugs, fitted as a linear trend continuous in dose; 95% CI = 95% confidence interval generally estimated using the profile likelihood, and when the profile CI did not converge were Wald-based; CED – cyclophosphamide equivalent dose. **Bound(s) cannot be estimated (No convergence after maximum iterations). LRT= likelihood-ratio test. ^a^P-values related to likelihood-ratio test resulting from extending the existing model (Radiation) by the additional specific types of chemotherapy (AA, CED, Anthracyclines, Platinum compounds, Epipodophyllotoxins, Vinca-alkaloids, Antimetabolites or specific cytotoxics agents received by at least 20 cases or controls: Procarbazine, Cyclophosphamide, Ifosfamide, Adriamycin, Carboplatin, Vincristine, Mercaptopurine and Thioguanine).

**Supplementary Table S8**: Evaluation of potential effect modifiers of the procarbazine dose-response

| **Characteristics** | **Mean cumulative procarbazine**  **dose, in g/m^2ψ^** | | **Cases / Controls** | **EOR/g/m^2^ [95% CI] of cumulative dose of procarbazine** |
| --- | --- | --- | --- | --- |
|  | **Cases** | **Controls** |  |  |
| **Sex** |  |  |  |  |
| Female | 7.2 (0.7-12.3) | 6.4 (4.3-7.8) | 10/7 | 0.39 [-0.02-4.04] |
| Male | 6 (0.1-18) | 4.6 (0.1-8.5) | 21/10 | 0.25 [0.00-1.29] |
| *P_interaction_* |  |  |  | *0.76* |
| **Age at Diagnosis of FPN (years)** |  |  |  |  |
| < 10 | 8.3 (0.7-18) | 5.4 (2-8.5) | 6/6 | 0.06 [-0.05-0.72] |
| 10–15 | 6.2 (0.1-12.3) | 6 (3.8-7.8) | 20/9 | 0.94 [0.10-10.45] |
| ≥ 15 | 4.7 (0.4-8) | 2.2 (0.1-4.3) | 5/2 | 0.28 [-0.11-10.03] |
| *P_interaction_* |  |  |  | *0.24* |
| **Decade of Diagnosis of FPN** |  |  |  |  |
| <1975 | 6.4 (0.7-11.8) | 4 (2-5.9) | 7/3 | 0.41 [0.07-4.61] |
| 1975–1985 | 7.9 (0.4-18) | 5.6 (3.8-7.8) | 17/11 | 0.27 [0.01-1.43] |
| ≥ 1985 | 1.9 (0.1-6.9) | 4.3 (0.1-8.5) | 7/3 | 0.03 [**-**] |
| *P_interaction_* |  |  |  | *0.20* |
| **Attained Age** **at STS (years)** |  |  |  |  |
| < 20 | 10.2 (3.9-18) | 5.8 (3.8-7.8) | 5/2 | 4709.26 [**-**] |
| 20–30 | 4.4 (0.1-11.7) | 4.7 (0.1-8.5) | 13/10 | 0.12 [-0.06-1.17] |
| ≥ 30 | 6.7 (1.1-11.8) | 5.3 (4.3-5.9) | 13/5 | 0.32 [-0.01-2.16] |
| *P_interaction_* |  |  |  | *0.37* |
| **Years from FPN Diagnosis (years)**^≠^ |  |  |  |  |
| < 15 | 6.3 (0.1-18) | 4.9 (0.1-7.8) | 17/8 | 0.77 [0.05-9.89] |
| 15–20 | 7.9 (4.9-11.8) | 6.4 (4.3-8.5) | 4/4 | 0.09 [-0.08-1.64] |
| ≥ 20 | 6 (1.1-10.7) | 4.5 (2-5.9) | 10/5 | 0.21 [-0.06-1.83] |
| *P_interaction_* |  |  |  | *0.61* |
| **Subsequent STS**^¥^ **by histological type** |  |  |  |  |
| Fibromatous neoplasms | 6.4 (0.7-18) | 4.1 (3.8-4.3) | 8/2 | 1.22 [0.02-36.45] |
| Myomatous and lipomatous neoplasms | 7.1 (4.5-10.7) | 6 (5.5-7.1) | 7/6 | 0.25 [-0.05-2.67] |
| Nerve sheath tumors | 8.4 (1.1-12.1) | 8.2 (7.8-8.5) | 6/3 | 0.19 [-0.06-8.39] |
| Soft-tissue tumors and sarcomas, NOS | 4.4 (0.1-12.3) | 3.3 (0.1-5.9) | 10/6 | 0.15 [-0.06-1.67] |
| *P_interaction_* |  |  |  | *0.28* |
| **Dose of radiation received to site of STS, mean in Gy** |  |  |  |  |
| 0 Gy | 6.6 (0.4-18) | 5.3 (3.8-7.8) | 5/7 | 0.06 [-0.05-1.06] |
| > 0-19.99 Gy | 6.3 (0.4-12.3) | 5.7 (2.0-8.5) | 18/8 | 0.39 [0.03-2.11] |
| >= 20 Gy | 6.6 (0.1-12.1) | 3.0 (0.1-5.9) | 8/2 | 0.66 [-0.01-16.52] |
| Missing | - | - |  |  |
| *P_interaction_* |  |  |  | ***<0.0001*** |

Abbreviations: FPN = first primary neoplasm; ^≠^Years from FPN Diagnosis = Years between FPN diagnosis and STS diagnosis; ^¥^STS = soft-tissue sarcoma [Kaposi’s sarcoma (n=8), synovial-like neoplasms (n=8), miscellaneous bone tumors (n=4), complex mixed and stromal neoplasms (n=3), blood vessel tumors (n=2), neurilemoma malignant (n=2), miscellaneous tumors (n=2), and others]; ^ψ^mean (minimum - Maximum). **Bound(s) cannot be estimated (No convergence after maximum iterations). EOR/ g/m^2^: Excess odds ratio of subsequent STS per g/m^2^ of cumulative procarbazine dose; 95% CI = 95% confidence interval generally estimated using the profile likelihood, and when the profile CI did not converge were Wald-based. *P_interaction_* from the likelihood ratio statistic test between model containing the linear trend in continuous cumulative procarbazine dose and categorical covariate in a loglinear term, and with that with the categorical covariate in an interaction with cumulative procarbazine dose.

**Supplementary Table S9**: Risk of subsequent soft-tissue sarcoma in relation to cumulative exposure to procarbazine adjusted for dose of anthracyclines and dose of radiation received to site of soft-tissue sarcoma.

| **Classes of cytotoxics** | **Mean dose (g/m^2^) in Cases / Controls** | **Mean years to STS** | **Cases / Controls** | **Odds ratio^$^**  **[95% CI]** |
| --- | --- | --- | --- | --- |
| **Procarbazine category** |  |  |  |  |
| 0 g/m^2^ |  | 23.1 | 234/248 | **1.0 (Reference)** |
| >0-6.00 g/m^2^ | 2.9/4 | 16.2 | 13/8 | 2.4 [0.8-8.4] |
| >6.00 g/m^2^ | 9.9/7.8 | 14.6 | 13/3 | 8.1 [1.7-77.8] |
| Missing |  | 22.7 | 15/16 |  |
| *P-value for trend* |  |  |  | ***0.04*** |
| **Anthracyclines category** |  |  |  |  |
| 0 g/m^2^ |  | 24.5 | 185/208 | **1.0 (Reference)** |
| >0-0.19 g/m^2^ | 0.1/0.1 | 14.9 | 21/16 | 2.1 [0.8-6.2] |
| 0.20-0.34 g/m^2^ | 0.3/0.3 | 17.0 | 22/23 | 0.9 [0.4-2.2] |
| >=0.35 g/m^2^ | 0.5/0.4 | 18.4 | 27/13 | 1.8 [0.7-4.7] |
| Missing |  | 21.5 | 20/15 |  |
| *P-value for trend* |  |  |  | *0.29* |

Abbreviations: 95% CI = 95% confidence interval. **^$^**Conditional logistic regression matched on sex, age at childhood cancer (± 1 year), calendar year of childhood cancer diagnosis (± 3 years), and retinoblastoma status (heritable/non-heritable).

**Figure S1**. Radiation dose–response relationship for risks of second primary soft-tissue sarcoma (STS) adjusted for Procarbazine. Odds ratios and corresponding 95% confidence intervals (circles), fitted linear excess odds ratio (EOR) per Gy (solid black line) and shaded lines are 95% confidence intervals of EOR.
